# Transaminase levels reflect disease severity in children ventilated for respiratory syncytial virus (RSV) bronchiolitis

**DOI:** 10.1038/s41598-018-20292-6

**Published:** 2018-01-29

**Authors:** Kentigern Thorburn, Crawford Fulton, Charlotte King, Difijah Ramaneswaran, Abdulaziz Alammar, Paul S. McNamara

**Affiliations:** 1 Paediatric Intensive Care, Alder Hey Children’s Hospital, Liverpool, L12 2AP UK; 2 Department of Clinical Infection, Microbiology & Immunology, The University of Liverpool, Liverpool, L69 7BE UK; 3 Department of Child Health, Institute in the Park (University of Liverpool), Alder Hey Children’s Hospital, Liverpool, L12 2AP UK; 4 Paediatric Respiratory Medicine, Alder Hey Children’s Hospital, Liverpool, L12 2AP UK

## Abstract

Bronchiolitis, often caused by respiratory syncytial virus (RSV), is the commonest cause of hospitalisation in infancy. Serum transaminases are sometimes raised in children with bronchiolitis. We tested the hypothesis that raised transaminases are associated with increased disease severity in children ventilated for bronchiolitis. Prospective observational cohort study of mechanically ventilated children with community-acquired RSV bronchiolitis. Alanine transaminase (ALT) and aspartate transaminase (AST) levels were measured daily. Children with normal transaminases were compared with those with elevated levels. Over 11 consecutive winters, 556 children with RSV bronchiolitis were mechanically ventilated – 226 had comorbidities and therefore excluded; 313 of remaining 330 were under 2 years age; 305 had early transaminase measurements. 57/305 (19%) had elevated transaminase (AST and/or ALT) levels. For the first time we show that duration of ventilation and length of admission were both significantly longer, and paediatric index of mortality and C-reactive protein higher, in those with elevated AST levels on admission (but not those with elevated ALT levels). Furthermore, transaminase elevations were transient, generally having normalised by seven days following admission. RSV bronchiolitis was more severe in children with early elevated AST levels and could be used early in the illness as a predictor for disease severity.

## Introduction

Respiratory syncytial virus (RSV) bronchiolitis causes significant morbidity and mortality worldwide every year^1,2^. RSV is the most common viral respiratory cause of hospital admission and of death in children below 5 years of age, especially in infancy^1^. RSV bronchiolitis occurs in predictable annual seasons (epidemics) that globally generate surges in hospitalisation rates, which often strain hospital and paediatric intensive care unit (PICU) capacity nationally and regionally. Approximately 3–10% of infants hospitalised with RSV bronchiolitis develop respiratory failure and require admission to PICU. In the United Kingdom between 2004 and 2012, bronchiolitis accounted for up to 12% of PICU admissions each year, with the vast majority (93%) being infants under 1 year of age^3^.

Despite most RSV research having concentrated on the lungs and the mechanics of pulmonary immunopathology, RSV bronchiolitis is not merely a single organ disease (i.e. lung) but impacts on extrapulmonary organ systems^4^. Elevated transaminases have been described in a subset of children who require mechanical ventilation on PICU with RSV bronchiolitis^5–7^. Suggested causes for this phenomenon include: virus-induced hepatitis (including RSV itself), high viral load leading to spill-over of RSV from the lungs into the systemic circulation, hepatotoxic medication, hypoxic hepatitis, hepatic congestion or ischaemia secondary to right heart failure/strain. With the advent of new anti-viral drugs for treatment of viral lower respiratory tract infections (LRTI), it will become important to differentiate infection-related transaminitis from medication-related hepatotoxicity. It has been suggested in previous smaller studies that children with raised transaminases have increased disease severity^5–8^. Comorbidity in this patient group (especially congenital heart disease and chronic lung disease) can confound interpretation of cause and effect – as the underlying comorbidity could play a role in the right ventricular failure and/or pulmonary hypertension.

To interrogate the hypothesis that children ventilated for RSV bronchiolitis with raised transaminases have increased severity of disease, we studied a novel homogenous cohort of children under 2 years of age over 11 RSV seasons/11-year period. To avoid non-RSV disease confounding interpretation, children with comorbidities were excluded from analysis.

## Methods and Measurements

### Objectives

Aim of the study was to compare disease severity as judged by duration of ventilation, length of PICU admission, respiratory indices and mortality in young children mechanically ventilated for RSV bronchiolitis with and without elevated alanine transaminase (ALT) and aspartate transaminase (AST) levels.

### Setting

21-bed regional multidisciplinary tertiary PICU in a university-affiliated, multispecialty, children’s hospital, which is also the regional paediatric cardiac referral centre, with an annual PICU admission rate of around 1100 children.

### Patients

All children under 2 years age mechanically ventilated with community-acquired RSV bronchiolitis over 11 consecutive RSV seasons (October 2002–March 2013) who had had liver function tests performed within 48 hours of admission to PICU were included. Children with any comorbidity (congenital heart disease, chronic lung disease, airway abnormalities, genetic anomalies, gastrointestinal abnormalities, immunodeficiency, central nervous system, neuromuscular abnormalities) and any nosocomial or healthcare-acquired RSV infection were excluded to remove non-RSV disease as a confounding factor for increased transaminase levels. The study was approved by the Clinical Audit Department – a division of the Directorate of Research and Clinical Development at the Alder Hey Children’s NHS Trust (reference number 5192). The Directorate ruled that institutional ethics review was not required as data were obtained from routine clinical investigations and records and patient details were not identifiable.

### Virology/Microbiology

Diagnostic samples of nasopharyngeal aspirates (for RSV detection) and lower airway secretions (for bacterial culture) through endotracheal tube using sterile precautions^9^ were taken on admission. Samples were collected by specialist respiratory physiotherapists or PICU staff members. Over the 11-year period, a number of different tests were used to detect RSV. Thus RSV was identified by immunochromatographic assays: Directigen^TM^ RSV test (2003–2004) or NOW^TM^ RSV test (2005–2013); or immunofluorescence: SimulFluor^TM^ reagents; and/or multiplex RT-PCR: Invitrogen^TM^ (2009–2013). Bacterial co-infection was defined as microbiologically proven bacterial infection – required bacteria colony counts >10^5^ cfu/ml of diagnostic sample for each single species obtained from lower airways secretions or urine, or a positive blood culture.

### Biochemistry

Most children had, as part of a daily routine biochemical profile, aspartate transaminase (AST) and alanine transaminase (ALT) levels determined, soon after admission to PICU and daily thereafter. Transaminase levels and C-reactive protein (CRP) were measured by photometric assays (Integra system^TM^, Roche Diagnostic Ltd.) at the laboratories for Clinical Chemistry at the Alder Hey Children’s Hospital. Normal and elevated paediatric reference ranges were based on those published by Hick and Soldin^10^. In infants <3 months of age AST and ALT levels were elevated when above 73 IU/L and 44 IU/L respectively. In infants and children >3 months of age, elevated AST and ALT levels were defined as being above 58 IU/L and 36 IU/L and respectively. To enable the analysis of liver function tests in children of different ages, transaminase levels were graded based on the Division of Microbiology and Infectious Diseases (DMID) paediatric toxicity tables (November 2007)^11^. Thus, Grade 1 was defined as 1.1 - < 2.0 × upper limit of normal (ULN) for age; Grade 2 as 2.0 - < 3.0 × ULN; Grade 3 as 3.0–8.0 × ULN; Grade 4 as >8.0 ULN. Given the small numbers of children, data from those with Grade 3 and 4 toxicity were analysed graphically together.

### Respiratory support

All forms of mechanical ventilation (conventional and high frequency) were carried out on PICU. Lung protection strategies, such as permissive hypercapnia and restriction of excessive tidal volumes (volutrauma), were standard practice. Inhaled nitric oxide was utilized in severe lung disease when hypoxaemia had been refractory to prior ventilator strategies and/or modes. Muscle relaxants and steroids were not routinely used.

### Data analysis

Prospective observational cohort study. Results were expressed as a number out of the total study population: mean and standard deviation (SD) or standard error (SE), or median and inter-quartile ranges (IQR). The Paediatric Index of Mortality (PIM) and resulting predicted probability of death were calculated from variables extracted on admission^12^. Markers of severity utilized were: duration of ventilation; length of PICU admission; worst oxygen index (mean airways pressure X FiO_2_/PaO_2_) and ventilation index (respiratory rate X PaCO_2_ X peak inspiratory pressure/1000) within the first 72 hours of PICU admission^13^; PIM^12^ and mortality. Continuous data were analysed using the Mann-Whitney-U test and categorical data using Fisher’s exact test. Statistical calculations were performed with the Statistical Program for Social Science release 21.0 (SPSS 21, Chicago, IL). A p-value < 0.05 was considered statistically significant. All p-values were two-tailed.

### Data availability

All data are available from the corresponding author on reasonable request.

## Results

Over the study period of 11 RSV seasons, 556 children with RSV bronchiolitis were mechanically ventilated – 226 had comorbidities and were therefore excluded; 313 of the 330 with no comorbidity were under 2 years of age. 305/313 (97%) had transaminases measured on either Day 1 (293 (94%)) or Day 2 (269 (84%)). Subsequently, 245 (78%), 205 (65%), 150 (48%), 94 (30%), 64 (20%) children had transaminases measured on Days 3, 4, 5, 6, and 7 respectively. Children generally only had transaminases measured when intubated and ventilated. The decreasing numbers of tests performed over time reflects that these children were improving and being extubated. 57 (19%) of the study group with no comorbidities (n = 305) had early elevated transaminase levels. The characteristics of this *no-comorbidity* study group are shown in Table 1. Most (95%) were under 1 year old and the majority (81%) less than 3 months. Only 3 patients died over the 11 RSV seasons *–* too low to differentiate between groups.

**Table 1:** Clinical and demographic characteristics of children ventilated with RSV bronchiolitis with and without elevated transaminase (AST and/or ALT) levels within 48 hours of PICU admission.

	Normal transaminase levels	Elevated transaminase levels (AST and/or ALT)
Number (male)	248 (140)	57 (32)
Median (IQR) age (months)	1.5 (1.5)	1.4 (2.3)
Number born ex-preterm (<37 weeks gestation: %)	90 (36)	12 (21)*
Number with bacterial co-infection (%)	80 (32)	18 (32)
Number died (%)	2 (1)	1 (2)

ALT = alanine transaminase; AST = aspartate transaminase; IQR = interquartile range; *p < 0.05.

Grading of transaminase elevation according to DMID criteria (paediatric toxicity tables)^11^ in age-group subgroups are presented in Table 2. All subsets (age and/or grade) were similar. There was no clear relationship between age and grade for either AST or ALT, but numbers were small in some subsets.

**Table 2:** Grading of transaminase elevation according to Division of Microbiology and Infectious Diseases (DMID) criteria (paediatric toxicity tables)^11^ based on age.

		AST (DMID grading)					ALT (DMID grading)				
		Normal	Grade 1	Grade 2	Grade 3/4	Total	Normal	Grade 1	Grade 2	Grade 3/4	Total
Age range (months) number [%]	0–3	224 [91.1]	15 [6.1]	1 [0.4]	6 [2.4]	248 [100]	210 [85.4]	15 [6.1]	7 [2.8]	14 [5.7]	246 [100]
	3–6	25 [92.6]	2 [7.4]	0 [0]	0 [0]	27 [100]	23 [85.2]	3 [11.1]	0 [0]	1 [3.7]	27 [100]
	6–12	10 [66.7]	4 [26.7]	0 [0]	1 [6.7]	15 [100]	11 [73.3]	2 [13.3]	0 [0]	2 [13.3]	15 [100]
	12–24	14 [82.4]	1 [5.9]	1 [5.9]	1 [5.9]	17 [100]	13 [76.5]	2 [11.8]	1 [5.9]	1 [5.9]	17 [100]
	Total	273 [89.5]	22 [7.2]	2 [0.7]	8 [2.6]	305 [100]	257 [84.3]	22 [7.2]	8 [2.6]	18 [5.9]	305 [100]

ALT = alanine transaminase; AST = aspartate transaminase.

**Figure 1:**
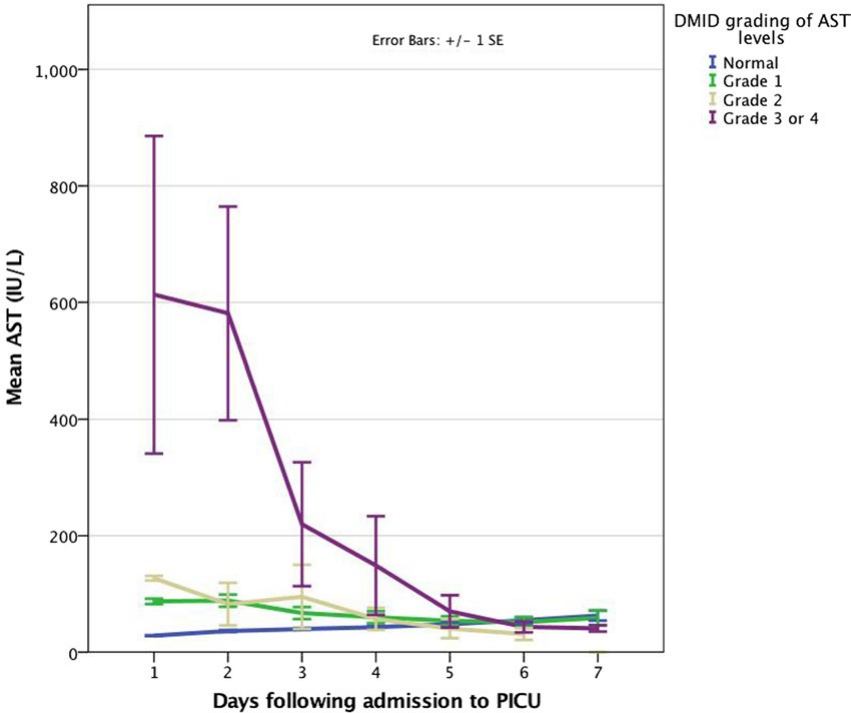
Changes in serum aspartate transaminase (AST) concentrations based on DMID grading^11^ following admission to PICU in children ventilated for RSV bronchiolitis.

**Figure 2:**
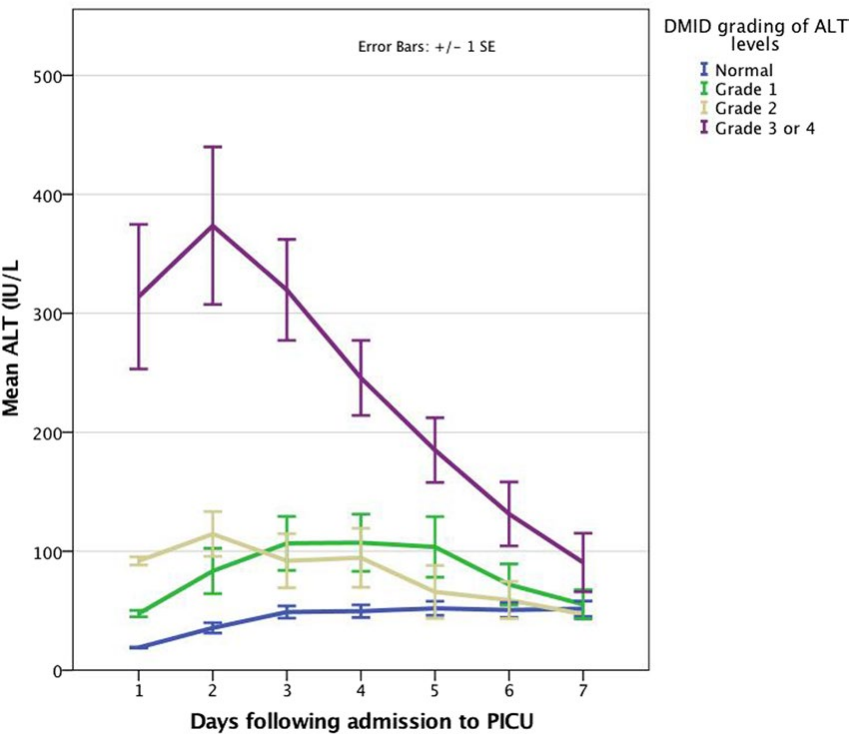
Changes in serum alanine transaminase (ALT) concentrations based on DMID grading^11^ following admission to PICU in children ventilated for RSV bronchiolitis.

Changes in serum AST and ALT concentrations (based on DMID grading) following admission to PICU are shown in Figs 1 and 2 respectively. AST levels were highest at admission and ALT levels peaked around day 2 to 3 of PICU stay. Elevations in transaminases were transient and generally returned to normal within a week, apart from those in the severest ALT subgroup (Figs 1 and 2).

Markers of disease severity in the subgroups are presented in Table 3. The duration of ventilation and length of PICU admission were both significantly longer in the group with elevated AST levels and they had a higher Paediatric index of mortality (PIM)^12^ score and CRP (all p < 0.05). Only PIM score was higher in those with elevated ALT levels. An AST/ALT ratio >2 was associated with prolonged ventilation, increased length of PICU admission and elevated CRP (Table 3).

**Table 3:** Markers of disease severity in children ventilated for RSV bronchiolitis with and without elevated transaminase (AST and ALT) levels within 48 hours of PICU admission – mean (standard deviation).

	AST		ALT		AST/ALT ratio	
	Normal (n = 273)	Elevated (n = 32)	Normal (n = 256)	Elevated (n = 48)	<2 (n = 227)	>2 (n = 65)
Length of ventilation (days)	4.9 (3.7)	6.9 (7.0)†	5.1 (4.4)	4.9 (3.0)	4.8 (3.5)	6.2 (6.1)*
Length of stay on PICU (days)	5.5 (4.0)	7.9 (7.0)*	5.7 (4.6)	6.1 (5.1)	5.5 (4.3)	6.7 (6.1)*
Worst oxygen index	9.6 (7.3)	10.1 (5.3)	9.6 (7.3)	9.7 (6.4)	9.7 (6.6)	9.5 (7.4)
Worst ventilation index	37.7 (29.0)	36.1 (21.5)	37.8 (29.1)	36.5 (23.8)	32.8 (21.2)	39.2 (30.2)
Paediatric Index of Mortality^12^	0.087 (0.069)	0.108 (0.059)*	0.086 (0.071)	0.103 (0.056)*	0.090 (0.062)	0.089 (0.071)
C-reactive protein (mg/L)	35 (40)	55 (52)*	34 (39)	50 (49)	32 (37)	56 (51)^†^

ALT = alanine transaminase; AST = aspartate transaminase; Mean (SD); *p < 0.05; ^†^p < 0.01.

Oxygen index (OI) = mean airways pressure X FiO_2_/PaO_2_ – worst OI within first 72 hours of PICU admission.

Ventilation index (VI) = respiratory rate X PaCO_2_ X peak inspiratory pressure/1000 – worst VI within first 72 hours of PICU admission.

## Discussion

This novel sizeable observational study of young children ventilated for RSV bronchiolitis supported the hypothesis that those with elevated transaminases, but only aspartate transaminase (AST), had increased severity of disease – as judged or measured by duration of mechanical ventilation, length of PICU admission and PIM. Raised transaminase levels were not uncommon in this patient group, occurring in nearly a fifth (19%) of our study population. This is the first study on this topic using a large and homogenous study cohort with community-acquired RSV bronchiolitis that is not ‘contaminated’ by underlying comorbidity or nosocomial infection.

Clinical characteristics were similar in children both with elevated and normal transaminases, bar previous prematurity in the elevated group. Additionally, no specific subset for age-group or grade stood out when categorised by DMID criteria (paediatric toxicity grading)^11^. Clinical impact appeared not to be associated with age-group or grade of transaminase elevation. The elevations in transaminases were transient with AST settling earlier than ALT, but both generally returning to normal ranges within a week (Figs 1 and 2). This would suggest a temporary phenomenon that followed a bronchiolitis-like time course rather than permanent or substantial injury, even at transaminase grade 3 or 4 toxicity levels.

Disease severity was significantly increased in the group with elevated AST levels on admission as measured by longer duration of ventilation and length of admission and higher PIM (risk of death on PICU admission) (p < 0.05), but not those with elevated ALT levels (Table 3). Interestingly CRP was also higher in this group (p < 0.05), suggesting an enhanced inflammatory state compared to the other groups. The aetiology for the elevated transaminases in our study group is not clear, but possible causes include: virus-induced hepatitis (including RSV itself), high viral load leading to spill-over of RSV from the lungs into the systemic circulation, hepatotoxic medication, hypoxic hepatitis, hepatic congestion or ischaemia secondary to right heart failure/strain, non-hepatic transaminases (muscle – heart and skeletal, kidney).

Despite RSV infecting the respiratory epithelial cells and predominantly causing lung disease, clinical impact peripheral to the lung parenchyma is described in RSV infection^4^. Extrapulmonary effects (like hepatitis or myocarditis) raise the question as to whether direct RSV effects (i.e. RSV infection of liver or myocardium) or indirect effects (secondary to parenchymal lung disease and its causative respiratory failure or due to circulating inflammatory mediators) are at play? RSV has been detected in the liver, myocardium, and cerebrospinal fluid^4^. However, most often it was RSV-RNA detected by RT-PCR, which is essentially cell-associated RSV genome and not necessarily viable RSV. It is yet to be conclusively demonstrated that RSV can escape the alveolar macrophages and replicate in distant organs. Our previous study discounted concomitant viruses causing hepatitis in this patient group^6^. It is unlikely that elevations in transaminases were due to drug-induced hepatotoxicity. This group of children are generally not prescribed medications in the community (apart from occasional courses of amoxicillin), as there are no specific treatments for bronchiolitis. Furthermore, no hepatotoxic medications were used following hospitalisation in both cohorts (elevated transaminases and normal levels) and elevations in transaminases were transient, even at transaminase grade 3 or 4 toxicity levels (Figs 1 and 2), making drug-induced hepatotoxicity improbable. It is unlikely that hypoxic hepatitis was the differentiating cause of elevated transaminases as both groups had similar oxygen indices (Table 3). Additionally, hypoxic hepatitis is mostly seen in severe hypoxia (arterial PO_2_ < 6kPa)^8,14^ whereas our patients’ oxygen levels were continuously monitored and hypoxia was avoided (oxygen saturations maintained above 92% in this patient group without congenital heart disease or chronic lung disease).

Myocardial dysfunction is well described in children with severe RSV bronchiolitis, especially those with congenital heart disease^15–18^ – hence their specific exclusion for this study. Previously, transient raised cardiac troponin levels (resolved by admission day 4) demonstrating myocardial injury or strain in children ventilated for RSV bronchiolitis with structurally normal heart was shown in up to 40% of this patient group^7,18^. No significant left ventricular dysfunction was identified on echocardiography in this patient group ventilated for RSV bronchiolitis^7,18^. However, reduced right ventricular function assessed by echocardiography (Tei index) was found in 20%, but not associated with raised cardiac troponin levels^7^. Neither was there an established association between reduced right ventricular function and elevated transaminases, but sample size was small (n = 34)^7^. Hepatic congestion or ischaemia secondary to right heart strain consequential to severe lung parenchymal disease is a plausible explanation for the elevated AST in our study. More severe lung disease in the group with elevated AST was attested by longer duration of mechanical ventilation and increased length of PICU admission. Right ventricular dysfunction/strain has been shown in children ventilated for RSV bronchiolitis with structurally normal hearts^7^. Fortunately, raised transaminase levels were transient, settling within 4–5 days, thereby making substantial or lasting (hepatic) injury less likely in our young study population.

Although transaminase activity is commonly regarded as a measure of liver function or hepatocellular injury, both ALT (cytoplasmic) and AST (cytoplasmic + mitochondrial) are also found in heart muscle, skeletal muscle and kidney^19^. The AST/ALT ratio (De Ritis ratio) is utilized as an indicator of the aetiology of hepatitis with a ratio <2 suggestive of (acute) viral hepatitis. AST/ALT ratios >2 are seen in newborns (especially following neonatal asphyxia), rhabdomyolysis and muscle injury, fulminant hepatitis or alcoholic hepatitis^19^. In this study AST/ALT ratio >2 was associated with prolonged ventilation, increased length of PICU admission and elevated CRP (Table 3). It is possible that the elevated AST accounting for this finding was generated from muscle injury or strain – cardiac and/or respiratory skeletal muscle. This group of patients did have more severe disease (prolonged ventilation and PICU admission) and right ventricular strain has been demonstrated in such children ventilated for RSV bronchiolitis^7^.

Predicted probability of death calculated from PIM scores was higher in children with elevated transaminase levels as compared to those with normal levels. This increased risk of death infers increased disease severity in the elevated transaminase group on admission and supports elevated transaminase levels as a potential additional marker of severity. Only 3 children died over the 11 RSV seasons – too low in any group for mortality to be utilised as a marker for severity.

Without a comparable non-RSV group, it is difficult to assess whether the association of elevated transaminases to severity of disease is a RSV-specific phenomenon or merely reflective of severity of lung parenchymal disease *per se*. Future studies should include patients with non-RSV lung disease. To establish whether elevated transaminase levels would predict severity of disease in non-ventilated children with RSV bronchiolitis will be more challenging. They are less likely to undergo routine blood sampling and reliable measurements of disease severity would need to be determined.

## Conclusions

RSV bronchiolitis was more severe in children admitted with elevated AST levels. This study suggests that early elevated AST levels could be used as a predictor for disease severity in RSV bronchiolitis.
